# Photodynamic Inactivation Potentiates the Susceptibility of Antifungal Agents against the Planktonic and Biofilm Cells of *Candida albicans*

**DOI:** 10.3390/ijms19020434

**Published:** 2018-02-01

**Authors:** Mu-Ching Huang, Mandy Shen, Yi-Jhen Huang, Hsiao-Chi Lin, Chin-Tin Chen

**Affiliations:** Department of Biochemical Science and Technology, National Taiwan University, Taipei 106, Taiwan; d02b22005@ntu.edu.tw (M.-C.H.); d03b22007@ntu.edu.tw (M.S.); r03b22001@ntu.edu.tw (Y.-J.H.); r04b22046@ntu.edu.tw (H.-C.L.)

**Keywords:** *Candida albicans*, antifungal drug, photodynamic inactivation, combination treatment

## Abstract

Photodynamic inactivation (PDI) has been shown to be a potential treatment modality against *Candida* infection. However, limited light penetration might leave some cells alive and undergoing regrowth. In this study, we explored the possibility of combining PDI and antifungal agents to enhance the therapeutic efficacy of *Candida albicans* and drug-resistant clinical isolates. We found that planktonic cells that had survived toluidine blue O (TBO)-mediated PDI were significantly susceptible to fluconazole within the first 2 h post PDI. Following PDI, the killing efficacy of antifungal agents relates to the PDI dose in wild-type and drug-resistant clinical isolates. However, only a 3-log reduction was found in the biofilm cells, suggesting limited therapeutic efficacy under the combined treatment of PDI and azole antifungal drugs. Using confocal microscopic analysis, we showed that TBO-mediated PDI could partially remove the extracellular polymeric substance (EPS) of biofilm. Finally, we showed that a combination of PDI with caspofungin could result in the complete killing of biofilms compared to those treated with caspofungin or PDI alone. These results clearly indicate that the combination of PDI and antifungal agents could be a promising treatment against *C. albicans* infections.

## 1. Introduction

*Candida albicans* is a major opportunistic fungal pathogen of humans [1]. This dimorphic yeast can cause tissue infections in the skin, mucosal oral cavity, gastrointestinal tract, vagina, and even the bloodstream of humans, especially in immunocompromised hosts [2]. Due to their similarities to mammalian cells, there are significant difficulties in developing new antifungal drugs. The mortality rate of patients with invasive candidemia is up to 40% every year even in patients that have received systemic antifungal treatment [3]. The risk factors of candidemia include catheter-related implantation, invasive surgeries, human immunodeficiency virus (HIV)-infection, cytotoxic chemotherapies, and the use of broad-spectrum antibiotics [4,5]. One major difficulty in managing infection is the ability of micro-organisms to attach to surfaces and develop resilient biofilms. *C. albicans* biofilms formed on mucosal surfaces and implanted on medical devices are associated with systemic infections and persistent infection [6,7,8]. The extracellular polymeric substance (EPS) found on the biofilms is considered to be a barrier to prevent the entrance of most commonly toxic agents [9,10]. Compared to planktonic cells, the ability to resist antifungal drugs increases 1000-fold in biofilms without specific drug-resistance genes [11]. Therefore, biofilms not only cause high mortality but also deteriorate the antifungal drug resistance. Fluconazole, a triazole antifungal agent, has been used in clinical settings to treat generalized fungus infections due to its reduced toxicity. However, an increasing incidence of drug resistance has been found in patients with frequent exposure to fluconazole [12]. Thus, management of *Candida* infection becomes a clinical challenge due to the increasing drug resistance and a shortage of effective antifungal agents.

Antimicrobial photodynamic inactivation (PDI) has been shown to be a potential approach for treating microbial infection induced by Gram-positive and Gram-negative bacteria as well as yeast. PDI is also considered to be an alternative approach for managing microbial strains with antibiotic resistance. PDI is a form of phototherapy involving visible light and a nontoxic photosensitizer (PS). When a PS is activated by a specific wavelength of light, the electron of the PS is excited from a ground state to a triplet state. The activated PS reacting with oxygen within and around its environment of micro-organisms can form singlet oxygen (^1^O_2_) or reactive oxygen species (ROS), which are toxic and cause cell damage and death rapidly by a nonspecific targeting effect [13]. Many published papers have shown PDI’s efficacy against various bacterial and yeast species [14,15,16]. Meanwhile, it has been shown that PDI is not only effective against drug-resistant strains but also shows no potential to develop drug resistance [17]. In addition, it has been shown that the combination of PDI with antifungal agents can increase the killing efficacy of planktonic *C. albicans* [18,19,20,21]. 

Previously, we have shown that the fungicidal effect of PDI could be augmented with chitosan in wild-type and clinical isolates of fluconazole-resistant *C. albicans* [22]. However, without PDI treatment, chitosan alone cannot exert significant toxicity against planktonic and biofilm cells of *C. albicans*. Further studies showed that the augmented cytotoxicity of chitosan relates to the level of PDI, suggesting that cellular damages were responsible for the fungicidal effect. In this study, we further investigated the ability of PDI to increase the susceptibility to antifungal drugs in the planktonic and biofilm cells of *C. albicans* as well as fluconazole-resistant clinical strains. We found that toluidine blue O (TBO)-mediated PDI combined with antifungal drugs results in a significant cytotoxicity against the planktonic and biofilm cells of wild-type and drug-resistant *C. albicans*. This study shows the possibility of using a lower dose of each agent to treat fungal infection and avert severe side effects.

## 2. Results

**Figure 1:**
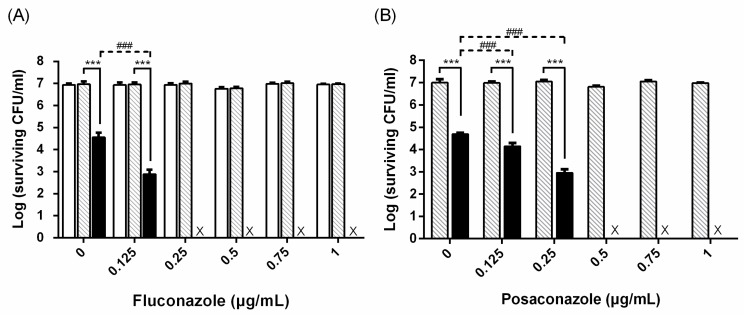
The cytotoxicity of combining photodynamic inactivation (PDI) and azole antifungal agents against planktonic *Candida albicans*. Cells were incubated with 0.1 mM toluidine blue O (TBO) and exposed to 50 J/cm^2^ of light. Following PDI, different concentrations of fluconazole (**A**) and posaconazole (**B**) were added and incubated for 24 h, then subjected to a plate count for measuring cell viability. ☐: PBS. 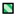: TBO only. ■: PDI. Each value is the mean obtained from three independent experiments ± standard deviation (SD), X represents the complete eradication of cells. *** *p* < 0.001 as compared to control, ### *p* < 0.001 as compared to PDI only.

To examine the susceptibility of PDI-treated *C. albicans* to antifungal drugs, we first examined the cytotoxicity induced by two azole drugs. As shown in Figure 1, no significant toxicity was found in the planktonic cell*s* of *C. albicans* under the treatment of 0.1 mM TBO or different concentrations of the azole drugs fluconazole and posaconazole. Meanwhile, a 2~3 log reduction was found in cells treated with 0.1 mM TBO plus 50 J/cm^2^ of light irradiation. The increase in cell killing results from the addition of different concentrations of fluconazole following TBO-mediated PDI (Figure 1A). A similar effect was also found by incubating with posaconazole (Figure 1B). Following PDI, the dose required for complete fungicidal killing by the addition of fluconazole or posaconazole was 0.25 and 0.5 μg/mL, respectively.

**Figure 2:**
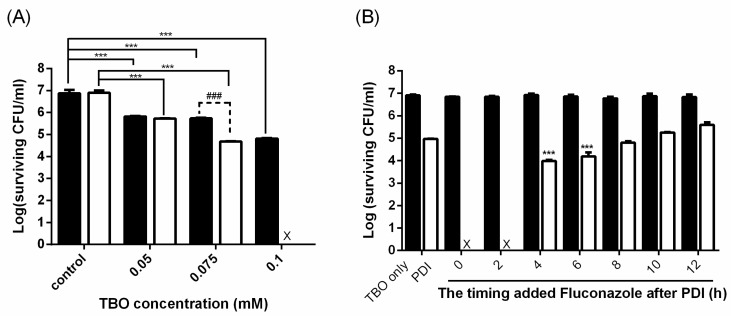
The cytotoxicity of combining PDI and fluconazole relates to the damage level induced by PDI. (**A**) *C. albicans* (10^7^ CFU/mL, colony-forming units per mL) was incubated with different concentrations of TBO and exposed to 50 J/cm^2^ of light. After PDI, 0.25 μg/mL fluconazole were added and incubated for 24 h, then subjected to a plate count for measuring cell viability. ■: PDI. ☐: PDI plus fluconazole. (**B**) Cells were incubated with 0.1 mM TBO and exposed to 50 J/cm^2^ of light. Fluconazole (0.25 μg/mL) was added at different time points post PDI and further incubated for 24 h, then subjected to a plate count. ■: TBO only. ☐: PDI. Each value is the mean obtained from three independent experiments ± SD, X represents the complete eradication of cells. *** *p* < 0.001 as compared to control, ### *p* < 0.001 as compared to PDI only.

We next examined whether the increased cytotoxicity relates to the damage level induced by PDI. As shown in Figure 2A, the increase in the cell killing relates to the PDI dose, which was shown in a TBO dose-dependent manner. Furthermore, we addressed the timing to add antifungal agents after PDI. As shown in Figure 2B, the planktonic cells of *C. albicans* were completely eradicated when fluconazole was added within the first 2 h following PDI. However, the effect of complete cell killing could not be found if fluconazole was added 4 h after PDI and the cytotoxicity gradually decreased. The increased cytotoxic effect of adding fluconazole could not be found 10 h post PDI. In other words, the PDI-induced fluconazole susceptibility against *C. albicans* became similar to that in untreated cells. These results implied that PDI-induced cellular damages could increase the susceptibility of *C. albicans* to antifungal drugs. However, in surviving *C. albicans*, the damages induced by PDI could be repaired over time, and the increased susceptibility to fluconazole treatment is then lost.

**Figure 3:**
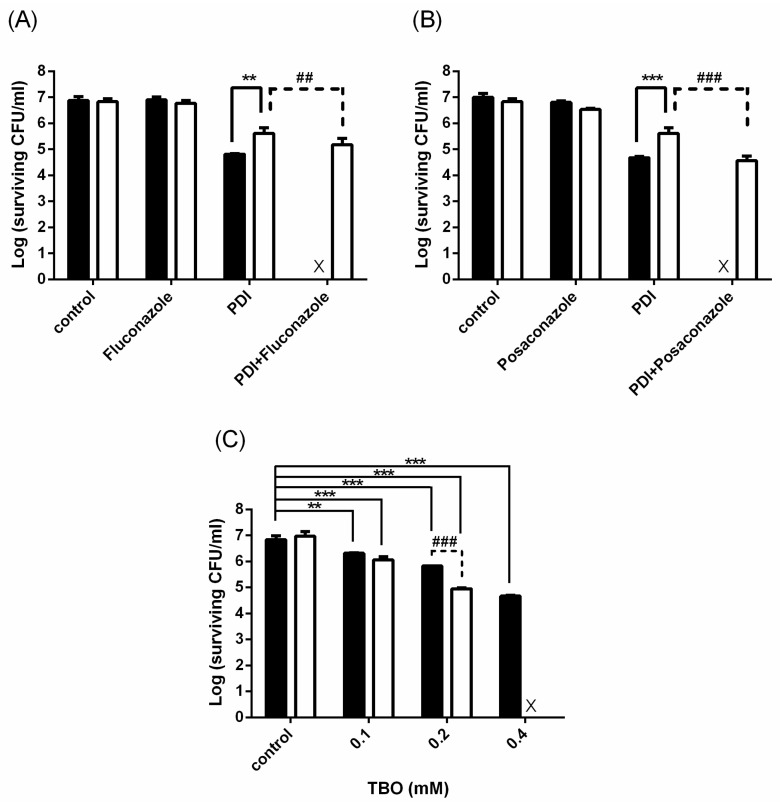
The cytotoxic effect of combining PDI and azole antifungal agents against a planktonic drug-resistant isolate of *C. albicans*. Cells (10^7^ CFU/mL) were treated with PDI (0.1 mM TBO plus 50 J/cm^2^ of light). After PDI, 0.25 μg/mL fluconazole (**A**) or 0.5 μg/mL posaconazole (**B**) was added and further incubated for 24 h. ■: Wild-type *C. albicans*, SC5314 ☐: *C. albicans* clinical isolate, 2008 no. 22. ** *p* < 0.01 *** *p* < 0.001 as compared to Wild-type, ## *p* < 0.01 ### *p* < 0.001 as compared to PDI only. (**C**) Drug-resistant cells were treated with different concentrations of TBO and exposed to 50 J/cm^2^ of light. Following PDI, 0.25 μg/mL fluconazole was added and further incubated for 24 h, then subjected to a plate count. ■: PDI only ☐: PDI plus fluconazole. Each value is the mean obtained from three independent experiments ± SD, X represents the complete eradication of cells. ** *p* < 0.01 *** *p* < 0.001 as compared to control, ### *p* < 0.001 as compared to PDI only.

To further elaborate our study, we examined whether the cytotoxicity of combining PDI with antifungal agents could be found against the clinical drug-resistant isolates of *C. albicans*. As shown in Figure 3A, PDI resulted in a 2-log and 1-log reduction in the wild-type strain and clinical isolate, respectively. After PDI, a complete cell killing could be found in the wild-type strain by incubating with fluconazole (Figure 3A) and posaconazole (Figure 3B). However, no increased cytotoxicity was found in the clinical drug-resistant isolate. As PDI could only exert a 1-log reduction in the clinical isolate, we speculate that the level of cellular damage might be not enough for the antifungal agent to act as shown in Figure 2. Therefore, we enhanced the PDI efficacy by increasing the TBO dose. As shown in Figure 3C, the combination of PDI and fluconazole could completely eradicate the drug-resistant isolates under the PDI condition of 0.4 mM TBO plus 50 J/cm^2^ of light irradiation. These results further support the concept that the increased susceptibility to antifungal agents relates to the damage levels induced by PDI.

**Figure 4:**
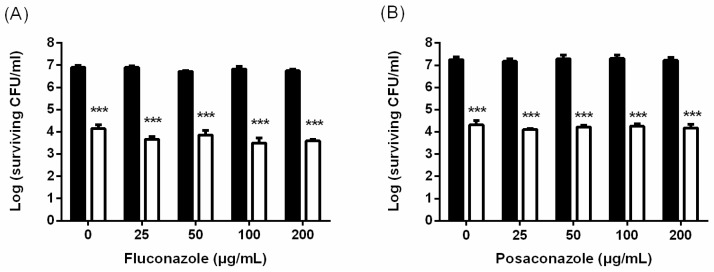
The effect of treatment combining PDI and an azole class of antifungal agents against *C. albicans* biofilms. Biofilm cells were incubated with 2.5 mM TBO and then exposed to 50 J/cm^2^ of light. After PDI, different concentrations of fluconazole (**A**) and posaconazole (**B**) were added and incubated for 24 h, then subjected to a plate count. ■: antifungal agents only. ☐: PDI plus antifungal agents. Each value is the mean obtained from three independent experiments ± SD. *** *p* < 0.001 as compared to control.

**Figure 5:**
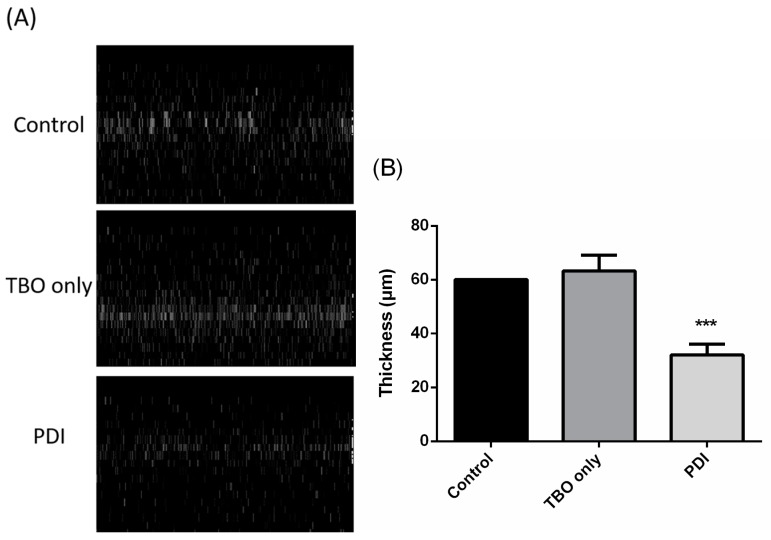
The thickness of biofilm extracellular polymeric substances (EPSs) in *C. albicans*. (**A**) Biofilms treated with PDI were subjected to a measurement of the thickness of EPS by using SYPRO^®^ Ruby Biofilm Matrix Stain. The fluorescence images were observed under a Leica SP2 confocal scanning fluorescence microscope. The Sagittal XZ section represents the biofilm’s thickness. (**B**) The quantified value of EPS thickness was measured by using the LAS AF lite software. The value of the EPS thickness is the mean from three independent experiments ± SD. *** *p* < 0.001 as compared to control.

It has been shown that the *C. albicans* biofilm is resistant to most antifungal agents due to the different phenotypic properties of biofilm cells and the dense and protected environment [23]. Therefore, we further examined whether the combination of PDI and antifungal drugs could exert a better eradiation against *C. albicans* biofilms. As shown in Figure 4, PDI could result in a 3-log reduction in biofilms; however, the addition of azole agents did not show the potentiating effect under elevated drug doses to 200 μg/mL. A unique biofilm property is produced by extracellular polymeric substances (EPSs) that provide shelter for *C. albicans* cells and hamper antifungal drug penetration. To elucidate the mechanism of losing susceptibility to azole drugs after PDI in biofilm, we therefore further examined the impact of PDI on the extracellular matrix by measuring the thickness of EPS in PDI-treated biofilms. As shown in Figure 5, the EPS thickness of the biofilms was significantly reduced after PDI, though they were not eradiated completely. Compared to the non-PDI groups, about half of the EPS was disrupted after PDI (2.5 mM TBO plus 50 J/cm^2^ of light dose), suggesting that PDI induced the disruption of biofilm EPS.

**Figure 6:**
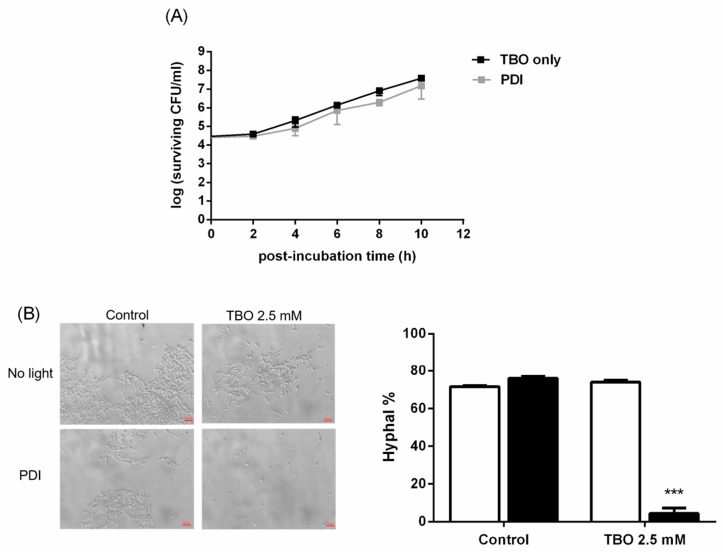
The degree of cell damage post-PDI in *C. albicans* biofilms. (**A**) The growth ability of biofilm cells was measured at different time points post PDI (2.5 mM TBO plus 50 J/cm^2^). Cells obtained from PDI-treated biofilms were suspended in YPD medium and subjected to a plate count. Each point is the mean of results obtained from three independent experiments and shown as mean ± SD. (**B**) Effect of PDI on hyphal formation in *C. albicans* biofilms. Cells obtained from PDI-treated biofilms were incubated in YPD medium supplemented with 10% FBS for 3 h. The ability of hyphal formation was observed and counted under microscope to determine the ratio of hyphal formation. The scale bar in the left panel is 20 µm. ☐: non-PDI. ■: PDI. Each value is obtained from three independent experiments and shown as the mean ± SD. *** *p* < 0.001 as compared to control.

Previously, we have shown that TBO could diffuse across the EPS and inside the biofilm [24]. We therefore further examined whether PDI could damage the biofilm’s cells by examining the growing ability and virulent ability. As shown in Figure 6A, biofilm cells without light irradiation enters the log phase at 2 h; however, cells obtained from PDI-treated *C. albicans* biofilms showed a slightly delay in entering the phase of exponential growth. These results indicate that PDI did not significantly affect the growth ability of *C. albicans* biofilm cells.

Yeast-to-hyphal switching is one of the major factors which govern the virulence in *C. albicans*. Thus, the morphological switching in biofilm cells treated with PDI was further examined by providing hyphae-inducing conditions. We first treated *C. albicans* biofilm cells with 2.5 mM TBO, then irradiated them with 50 J/cm^2^ of light dose. After PDI, biofilm cells were dispersed and grown in yeast peptone dextrose (YPD) medium containing 10% FBS to examine their filamentous ability. As shown in Figure 6B, the morphological switching ability of *C. albicans* was completely suppressed in biofilm cells treated with PDI, suggesting that some of the biological functions of biofilm cells were affected by PDI.

**Figure 7:**
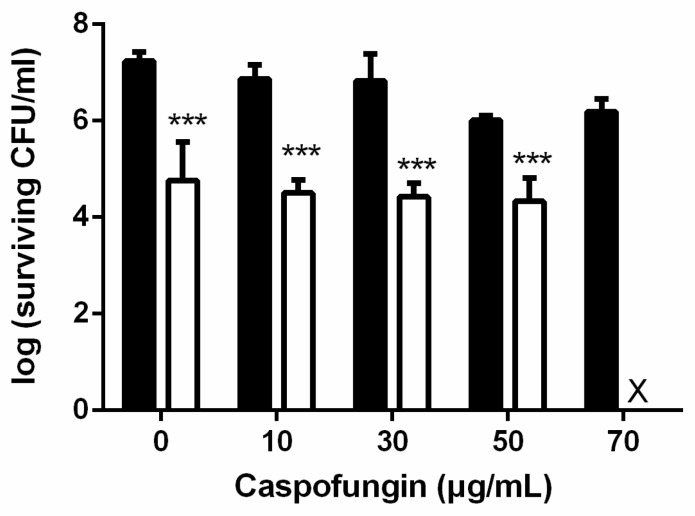
The antifungal activity against *C. albicans* biofilms by combining PDI and echinocandin. Biofilm cells were incubated with 2.5 mM TBO and exposed to 50 J/cm^2^ of light. After PDI, different concentrations of caspofungin were added to the cells, which were further incubated for 24 h and then subjected to a plate count. ■: caspofungin only. ☐: PDI plus caspofungin. Each value is the mean obtained from three independent experiments and shown as mean ± SD, X represents the complete eradication of cells. *** *p* < 0.001 as compared to control.

It has been reported that *Candida* cells in the biofilm environment are up to 1000-fold more azole-resistant than planktonic cells [25,26], suggesting that azole drugs are ineffective against the *Candida* biofilm. Compared to azole-type drugs, *Candida* biofilms are more sensitive to the echinocandin type of antifungal drug [27,28]. We therefore further examined the antifungal toxicity by combining PDI and caspofungin, an echinocandin antifungal agent. Within the range of 10 to 50 μg/mL caspofungin, no increased cytotoxicity was found in the biofilm cells pretreated with PDI. However, a successful eradiation of *C. albicans* biofilms could be found by incubating with 70 μg/mL of caspofungin following TBO-mediated PDI (Figure 7).

## 3. Discussion

Presently, azoles, polyenes, allylamines, and echinochandins are the major antifungal drugs used in clinic to treat *Candida* infection. However, the prevalence of drug-resistant *C. albicans* strains isolated in the hospital are increasing, especially those with resistance to azole-class antifungal drugs [29]. In addition, these antifungal drugs cannot fully meet the clinical requirements of adequate antifungal therapy due to their enunciated side effects [30]. Unfortunately, the development of antifungal drugs has not kept up with newly emerging drug-resistant strains. To resolve this complicated infection, the combination of different antifungal drugs have been used to treat *Candida* infection; however, no significant improvement and even an antagonistic effect has been found [31]. Therefore, new alternative antifungal approaches have been developed [32,33,34].

The increased therapeutic efficacy of combining PDI and antibiotics has shown its potential in treating bacterial infection [35,36,37]. In the study of Cassidy et al., a synergistic bactericidal effect was found by combining PDI and antibiotics used in treating Cystic Fibrosis pulmonary infection caused by *B. cepacia* complex strains [35]. In this study, we found that PDI can increase the susceptibility of drug-resistant clinical isolates and even biofilms of *C. albicans* to antifungal drugs. Under the conditions of our studies, antifungal agents alone had relatively little fungicidal activity at the concentration used. However, the combination of PDI and a lower dose of antifungal drugs could augment the cytotoxicity against the planktonic and biofilm cells (Figure 1). These studies indicate the feasibility of combining PDI and antimicrobial drugs for treating microbial infection. Using an SEM analysis, Cahan et al. demonstrated significant damage to the cell wall in *Escherichia coli* and *Staphylococcus aureus* treated with photosensitizer-antibiotics conjugates [36]. Poto et al. further showed that the increased bacterial killing of combining PDI and an antibiotic relates to the PDI-induced alteration in the biofilm structure of *S. aureus* [37]. In eukaryotic microbial cells, Giroldo et al. have shown that methylene-blue-mediated PDI could increase the membrane permeability of *C. albicans* [38]. Our previous study showed that the cell wall of *C. albicans* became fragmented after PDI [22]. Furthermore, a reduced EPS thickness was found in the biofilms of *C. albicans* treated with PDI as shown in Figure 5 of this study. Therefore, the increased susceptibility to antimicrobial drugs might relate to the nonspecific damage of the cell wall and the breakdown of biofilm matrix induced by PDI, which results in the increased drug uptake.

In this study, we observed that the planktonic cells of *C. albicans* treated with PDI only had a temporary increase in their susceptibility to antifungal drugs and the extent of this susceptibility relates to the PDI dose (Figure 2). Previously, we have shown that higher amounts of TBO are required for antifungal activity against drug-resistant strains compared to those of wild-type *C. albicans* [22]. In this study, we further showed that higher amounts of TBO are required for an increased susceptibility to azole-class agents in a drug-resistant clinical isolate (Figure 3). In addition, we further demonstrated that cells that have survived PDI could recover from the PDI-induced damage and lose their susceptibility to the lower dose of antifungal drugs. In this regard, an appropriate design of the treatment dose and schedule are necessary to achieve satisfactory therapeutic outcomes.

Previously, Kato IT et al. reported that the planktonic cells of *C. albicans* that have survived PDI have reduced virulence factors [20]. They found that cells that have survived sublethal PDI significantly reduced their ability to form germ tubes, a represented virulence factor, and that the reduction was more significant with a higher light fluence. In the present study, we found that the exponential growth was similar between the treated and untreated biofilm cells. However, alterations in the hypha formation were found in the PDI-treated biofilm cells (Figure 6B), suggesting that cell damage was induced in these biofilm cells. These results clearly justify the combined use of PDI and antifungal drugs to treat fungal infection.

*C. albicans* biofilms are notorious for their removal difficulty and resistance toward antifungal agents. Within biofilms, EPS plays a crucial role in providing a favorable environment, not only acting as a physical barrier which protects *C. albicans* from an antifungal drug attack [39], but largely modifying the behavior and gene expression of *C. albicans* associated with drug resistance [11]. In this study, we found that PDI could partially destroy the biofilm EPS (Figure 5). However, fluconazole was still not effective against the PDI-treated biofilm (Figure 4). It has been shown that *C. albicans* biofilms have a higher ability to resist fluconazole [40]. Nett et al. [41] further demonstrated that β-1,3-glucans, the major component of EPS in *C. albicans* biofilms, could sequester fluconazole. In this regard, the action of fluconazole might still be hindered by the remaining EPS. On the other hand, caspofungin belongs to the echinocandins group of antifungal agents, which has shown promising efficacy in treating *C. albicans* infections [42]. There have also been also reports that found that caspofungin has potential to combat biofilm-related conditions. Although fluconazole is not effective against biofilm, PDI indeed significantly increases the antifungal activity of caspofungin, which results in complete eradiation against the biofilms of *C. albicans* (Figure 7).

## 4. Materials and Methods

### 4.1. Candida Strains and Growth Conditions

The wild-type strain, SC5314 (ATCC MYA-2876D), was kindly provided by L.Z. Den, Department of Medical Technology, National Taiwan University, Taipei, Taiwan. The fluconazole-resistant clinical *C. albicans* strain, 2008 no. 22, was obtained from the infection control laboratory at the National Taiwan University Hospital, Taipei, Taiwan. *C. albicans* cells were grown in 10 mL yeast peptone dextrose (YPD) (Difco, Detroit, MI, USA) medium under aerobic conditions for 16 h at 37 °C. Cell suspensions were harvested following centrifugation, washed by phosphate-buffered saline (PBS; pH 7.4) three times, and re-suspended in PBS containing 10^7^ CFU/mL cells for the following PDI study.

### 4.2. Biofilm Preparation

*C. albicans* Biofilms were modified according to our published protocol [22]. Briefly, 10^7^ CFU/mL of cells were suspended in YPD medium and incubated in 48-well culture dishes, which contain removable 316 L stainless steel disks (0.6 cm in diameter). The cells were allowed to attached onto the disks for 1.5 h at 37 °C, and then the disks were removed to a new culture dish containing fresh YPD medium and further incubated for 48 h at 37 °C.

### 4.3. Treatment of PDI and Antifugal Agents in Planktonic and Biofilm Cells

In the planktonic studies, approximately 1 × 10^7^ CFU/mL of cells were incubated with different concentrations of TBO (Sigma-Aldrich, St. Louis, MO, USA) for 30 min at 25 °C and then shacked (100 rpm) in the dark. Then, cells were washed once by PBS and resuspended in PBS. After that, 200 μL samples were added to 96-well culture dishes (Costar; Corning Life Sciences, Lowell, MA, USA). A homemade high-power LED array (wavelength 630 ± 5 nm, 30 mW) was used to deliver 50 J/cm^2^ of light irradiation. Following PDI, cells were further incubated with different concentrations of fluconazole (Sigma-Aldrich, St. Louis, MO, USA) and posaconazole (Merck, Whitby, ON, Canada) in PBS for 24 h at 37 °C. For analyzing cell viability, cells were serially diluted 10-fold with PBS from 10^−1^ to 10^−5^ times. Each dilution was incubated on YPD agar plates at 37 °C for 18 h, then the colonies were counted and expressed as CFU.

In the biofilms studies, biofilms were incubated with different concentrations of TBO for 30 min at 25 °C in the dark. Disks containing biofilms were then moved to a new plate containing PBS and irradiated with the high-power LED array at 50 J/cm^2^. After PDI, cells were incubated with fluconazole, posaconazole, and caspofungin (Sigma-Aldrich, St. Louis, MO, USA) in PBS for 24 h at 37 °C. Then, disks were placed into test tubes containing 1 mL PBS and vortexed. The resulting *C. albicans* suspensions were serially diluted as described above for colony counting.

### 4.4. Growth Curves in PDI-Treated Biofilm

To examine the growth curve in PDI-treated biofilm cells, disks containing biofilms were placed into test tubes containing 1 mL YPD medium and vortexed. Then, 11.5 mL YPD medium was added, further incubated at 37 °C, and shacked at 150 rpm for different periods of time. At each time point, 1 mL of sample was removed and serially diluted 10-fold with PBS from 10^−1^ to 10^−5^ times, and each dilution was incubated on YPD agar plates at 37 °C for 18 h. The colonies were counted and expressed as CFU.

### 4.5. EPS Staining in Biofilms

To measure the thickness of biofilm EPSs, the EPS of biofilms treated with or without PDI was incubated with SYPRO^®^ Ruby Biofilm Matrix Stain (Invitrogen Corporation, Grand Island, NY, USA) for 1 h and then washed once by PBS. The fluorescence emission of a biofilm’s EPS was observed under a Leica SP2 confocal scanning fluorescence microscope (Leica Inc., Malvern, PA, USA) equipped with a 20× water-dipping objective lens. The fluorescence images were recorded upon excitation by a 488 nm diode laser and the emission was measured at 630 ± 70 nm. The biofilm thickness was obtained by converting the z-stack images of the biofilms. The quantified value of a biofilm’s EPS thickness was measured by using the LAS AF lite software integrated into the Leica SP2 confocal microscope.

### 4.6. The Ratio of Hyphal Formation

To examine the hyphal formation in biofilm cells, disks containing biofilms treated with or without PDI were placed into test tubes containing 1 mL YPD medium containing 10% FBS (Thermo Fisher Scientific, Waltham, MA, USA) and vortexed. The collected cell suspension was incubated at 37 °C and shacked at 150 rpm for 3 h. Then, the hyphal was observed under an Eclipse Ti inverted microscope (Nikon Instruments Inc., Melville, NY, USA).

### 4.7. Statistical Analysis

All results were obtained from three independent experiments and each value was expressed as mean ± SD. The two-tailed Student’s *t*-test was used to count whether the differences between two means were significant. *p* < 0.05 was considered significant.

## 5. Conclusions

PDI-induced damage can significantly increase the susceptibility to antifungal drugs of the *C. albicans* wild-type strain as well as its drug-resistant clinical isolate. This study indicates that the combined use of PDI and antifungal agents is a promising strategy in treating *C. albicans* infections.
